# Mitogenome structure and phylogenomics of the order Thoreales (Nemaliophycidae, Rhodophyta)

**DOI:** 10.1111/jpy.70207

**Published:** 2026-07-23

**Authors:** Monica O. Paiano, Morgan L. Vis, Nadia M. Lemes da Silva, John A. West, Shiva K. Rai, Javier Carmona‐Jiménez, Orlando Necchi

**Affiliations:** ^1^ Biological Sciences Department São Paulo State University São José do Rio Preto SP Brazil; ^2^ Environmental and Plant Biology Department Ohio University Athens Ohio USA; ^3^ School of Biosciences University of Melbourne Parkville Victoria Australia; ^4^ Department of Botany, Degree Campus Tribhuvan University Biratnagar Nepal; ^5^ Departamento de Ecología y Recursos Naturales, Facultad de Ciencias Universidad Nacional Autónoma de México, Ciudad Universitaria Coyoacán Ciudad de México Mexico; ^6^ Biological Sciences Department São Paulo State University, Rua Cristóvão Colombo, 2265 São José do Rio Preto SP Brazil

**Keywords:** freshwater red algae, mitochondrial genome, *Nemalionopsis*, phylogenomics, *Thorea*

## Abstract

Organelle genomes offer a powerful tool for red algal evolutionary studies. Although the number of Rhodophyta genomes has increased substantially over the past 2 decades, only two mitochondrial genomes have been reported for the red algal order Thoreales. In this study, we generated 10 new complete mitochondrial genomes of Thoreales, representing the order's two genera and eight species, which, combined with additional mitogenomes of related orders of the subclass Nemaliophycidae, resulted in a comprehensive dataset of 20 mitogenomes. Thoreales mitogenomes exhibited little variation, ranging in length from 25,022 bp to 26,369 bp, with GC content from 25.6% to 28.3%, gene numbers from 46 to 54, and protein‐coding genes from 21 to 25. Four genes were missing in one or more species (*atp*4, *rpl*20, *sdh*2, and *sdh*4), and no introns were detected. Synteny among members of Thoreales was highly conserved and similar to other orders of Nemaliophycidae. Phylogenomic analyses strongly supported the monophyly of Thoreales within Nemaliophycidae. Within the order, *Nemalionopsis* and *Thorea* as well as the relationship among the species received full support. *Nemalionopsis shawii* and *N. parkeri* formed an early‐diverging clade that was sister to *Thorea*, which had species that resolved into two distinct lineages lacking evident geographic or morphological differentiation. By expanding the available dataset from two to 10 mitogenomes, this study provides a broad organellar perspective on Thoreales evolution, contributing to new insights into the systematics and diversification of freshwater red algae and, potentially, for clarifying inter‐ordinal relationships within Nemaliophycidae.

**Abbreviations**
bp: base pairs
CCAP: Culture Collection of Algae and Protozoa
CDSs: coding sequences
LCB: locally collinear block
ML: maximum likelihood
NGS: Next‐Generation Sequencing

## INTRODUCTION

The red algae (Rhodophyta) are an ancient and diverse group of ca. 7750 photosynthetic species inhabiting mostly marine environments, with Florideophyceae being by far the most species‐rich class (Guiry & Guiry, 2026). Although several freshwater representatives are known within Rhodophyta, the subclass Nemaliophycidae is unique in comprising multiple lineages that have independently diversified in freshwater habitats. Currently, Nemaliophycidae includes 12 orders, of which three—Balbianiales, Batrachospermales, and Thoreales—are composed exclusively of freshwater taxa (Guiry & Guiry, 2026; Vis & Necchi, 2021). These exclusively freshwater orders have been the focus of single and multigene phylogenetic investigations aimed at clarifying infraordinal relationships (Costa et al., 2016; Entwisle et al., 2009; Lam et al., 2016; Müller et al., 2002; Saunders et al., 2018; Sheath & Müller, 1999).

The order Thoreales was formally proposed to accommodate the genera *Thorea* and *Nemalionopsis*, which had previously been placed within Batrachospermales (Müller et al., 2002). The recognition of Thoreales as a distinct order was supported by the combination of morphological traits and molecular evidence (Kumano, 2002; Vis & Necchi, 2021). The two genera are readily distinguished morphologically: *Nemalionopsis* possesses a mucilaginous matrix and reproductive structures located near the tips of the assimilatory filaments, whereas *Thorea* lacks a mucilaginous matrix and bears reproductive structures at the base of the assimilatory filaments. *Nemalionopsis* currently comprises only two species, whereas 12 species are recognized in *Thorea* (Vis & Necchi, 2021). Further morphological details and diagnostic characters with illustrations are provided in the relevant revisions on Thoreales (Johnston et al., 2018; Vis & Necchi, 2021).

Species of Thoreales exhibit a broad but disjunct geographic distribution, occurring in freshwater systems across multiple continents, including North America, South America, Europe, Asia, and Oceania. Records span temperate and tropical regions, with *Nemalionopsis* reported from North America and South Asia, and *Thorea* species documented from the Americas, Europe, Asia, and Pacific islands (Johnston et al., 2018; Vis & Necchi, 2021). Despite these clear generic distinctions, phylogenetic relationships within Thoreales and its placement relative to other Nemaliophycidae orders remain only partially resolved, as previous phylogenetic studies were based on single‐locus or limited multigene datasets (Johnston et al., 2018; Vis et al., 1998; Vis & Sheath, 1999). In this context, mitogenomic data can provide a comprehensive set of molecular characteristics that improve phylogenetic resolution and support robust inferences of evolutionary relationships across this disjunct distribution. The order Thoreales, which comprises 14 recognized species (Vis & Necchi, 2021), is the second most species‐rich group among the exclusively freshwater Nemaliophycidae, after the Batrachospermales. The genus *Nemalionopsis* has been shown as the earliest divergent lineage within the order in all phylogenetic studies, but this positioning requires testing based on a phylogenomic analysis.

Organellar genomes offer a powerful tool for red algal evolutionary studies. The genomic era has transformed algal research by enabling routine recovery of complete organellar genomes and delivering markedly improved phylogenetic resolution across multiple ranks (Costa et al., 2016; Díaz‐Tapia et al., 2017; Iha et al., 2018; Lemes da Silva et al., 2025). Dedicated databases, maintained by specialists in red algal biology, collect genome and transcriptome data from multiple studies and make them available through integrated analytical platforms, thereby facilitating comparative genomic research (Chen et al., 2018; Lipinska et al., 2023). In turn, broad phylogenomic datasets spanning mitochondrial and plastid genomes are revealing cryptic diversity, supporting formal reclassifications, and enabling the recognition of novel lineages (Díaz‐Tapia et al., 2019; Park et al., 2023; Yang et al., 2015). Comparative analyses of mitochondrial and plastid genomes across Nemaliophycidae have shown both a conserved core and lineage‐specific rearrangements, underscoring their value for phylogenomics and genome evolution (Lee et al., 2016; Salomaki & Lane, 2016). Comparable studies in the closely related order Batrachospermales have already demonstrated the potential of mitochondrial genomes for resolving evolutionary relationships and characterizing genome architecture (Lemes da Silva et al., 2025; Paiano et al., 2017, 2018), providing a relevant framework for extending similar approaches to Thoreales.

Despite the increasing availability of organellar genomes across red algae, sampling within Thoreales remains sparse and is currently restricted to a single species, *Thorea hispida*, which has only two mitochondrial and three chloroplast genomes published to date (Cho et al., 2018; Nan et al., 2017, 2020). Expanding organellar genome coverage across the order may provide valuable insights into the evolutionary relationships of Thoreales.

The aims of this study were (i) to characterize the structural features of the mitogenomes in the order Thoreales, comparing the patterns within the order and with other Nemaliophycidae orders and (ii) to infer phylogenetic relationships based on a representative taxon sampling, to provide new insights into mitochondrial genome evolution within Thoreales and contribute to resolving phylogenetic relationships in the Nemaliophycidae.

## MATERIALS AND METHODS

### Taxon sampling, genomic DNA isolation and next‐generation sequencing

Ten samples comprising both genera, *Nemalionopsis* and *Thorea*, of the freshwater red algal order Thoreales were sequenced: the two known *Nemalionopsis* species and eight samples of six *Thorea* species. Collection details are in Table S1. For *N. parkeri*, a cultured strain (voucher CCAP 1376/1) was obtained from the Culture Collection of Algae and Protozoa (CCAP, www.ccap.ac.uk) and used for genomic DNA extraction and sequencing. Details on the original collections are provided in Table S1. Specimens used were divided in two portions: (i) for DNA sequencing by immediately being placed in silica gel and (ii) for vouchering by either liquid preservation or pressed on herbarium paper and deposited in recognized herbaria (Thiers, 2026; Table S1). All specimens were previously identified based on current diagnostic characters using appropriate taxonomic literature (Kumano, 2002; Müller et al., 2002; Vis & Necchi, 2021) and checked for sequence identity using the COI‐5P gene barcode region against sequences in the GenBank database.

Three extraction methods were used. For *Thorea gaudichaudii*, *T. indica*, and *T. kokosinga‐pueschelii*, the material was homogenized in a bead‐beater and a modified (cetyltrimethylammonium bromide) extraction protocol with polyvinylpyrrolidone‐10 was utilized (Schenk et al., 2023). For *Nemalionopsis parkeri, N. shawii*, *T. bachmannii*, two specimens of *T. hispida* (Great Britain and Spain), and *T. riekei* (Mexico), genomic DNA (gDNA) was extracted using the Qiagen MagAttract High Molecular Weight Kit (Qiagen, Hilden, Germany) following the manufacturer's protocol. For *T. riekei* (Texas), genomic DNA was extracted using the Qiagen DNeasy PowerPlant Pro Kit following the manufacturer's protocol. For all extractions, the gDNA was quantified using a Qubit 2.0 fluorometer (ThermoFisher Scientific, Waltham, Massachusetts, United States) and Quant‐it dsDNA High‐Sensitivity Assay kit. Samples were immediately sent to Life Sciences Core Facility (LaCTAD) from the State University of Campinas (UNICAMP) for quality checking in a TapeStation 4200 system (Agilent, Santa Clara, United States). Libraries were prepared with the Illumina DNA prep protocol and sequences on an Illumina NextSeq 2000 platform (Illumina, San Diego, California, United States) with paired‐end 2 × 150 bp (base pairs).

### Mitogenomes assembling and annotation

For the 10 samples, the Illumina reads were checked for quality with Fast QC (v0.12.1; Andrews, 2016) and trimmed with Trimmomatic version 0.39 (Bolger et al., 2014) to remove adapters and low‐quality reads (*Q* < 20), with the following parameters: ILLUMINACLIP: adapters.fa: 2:30:10; SLIDINGWINDOW: 4:20; MINLEN: 36. Mitogenomes were de novo assembled using different assemblers: NOVOPlasty version 4.3.1 (Dierckxsens et al., 2017), with the *Thorea hispida* (KY083066) mitogenome sequence in the assembly as the seed sequence and k‐mer 29 and insert size 300; GetOrganelle version 1.5.1c (Jin et al., 2020), with k‐mers 21, 45, 65, 85, 105, ‐R 100, ‐F other _pt; and SPAdes version 3.15.5 (Nurk et al., 2017; Prjibelski et al., 2020), depending on the specificities of each sample. For *T. gaudichaudii*, the mitogenome did not circularize following de novo assembly. The published mitochondrial genome of *T. hispida* (KY083066) was used as a reference to identify the mitochondrial contigs and close the gaps, resulting in a circularized genome assembly. Geneious Prime® 2025.1.3 (Biomatters, https://www.geneious.com) was used to iteratively map trimmed reads to extend the contigs, with low sensitivity and a minimum mapping quality of 30 until complete mitochondrial genome sequences could be circularized.

Genome annotation was performed by MFannot (Beck & Lang, 2010), and the tRNAs were identified with tRNAscan‐SE version 2.0.7 annotator in GeSeq with default parameters (Chan & Lowe, 2019). All annotations were carefully reviewed and manually corrected in Geneious Prime® 2025.1.3. Physical maps of the 10 mitochondrial genomes generated in this study were prepared using OGDRAW v1.3.1 (https://chlorobox.mpimp‐golm.mpg.de/OGDraw.html; Greiner et al., 2019; Figure S1).

### Phylogenomic analysis

A total of 20 sequences of mitochondrial genomes were used in the phylogenetic analyses: 12 Thoreales, 10 generated in this study, and two retrieved from GenBank. Eight mitogenome sequences of Nemaliophycidae were used as outgroups representing the orders Balbianiales, Batrachospermales, and Palmariales (Figure 1). Sixteen coding genes (*atp*6, *atp*9, *cob, cox*1, *cox*2, *cox*3, *nad*1, *nad*2, *nad*3, *nad*4, *nad*4L, *nad*5, *nad*6, *rpl*16, *rps*11, *rps*12) present in all 20 taxa were used in the analyses. These genes were manually concatenated and aligned in Geneious Prime® 2025.1.3 at the nucleotide level using the MUSCLE v5.1 algorithm (Edgar, 2022). The final alignment was 14,386 bp in length. A maximum likelihood (ML) phylogenetic tree inferred from the nucleotides was obtained with RAxML version 8 (Stamatakis, 2014), using 1000 fast bootstraps in the parameters.

**FIGURE 1:**
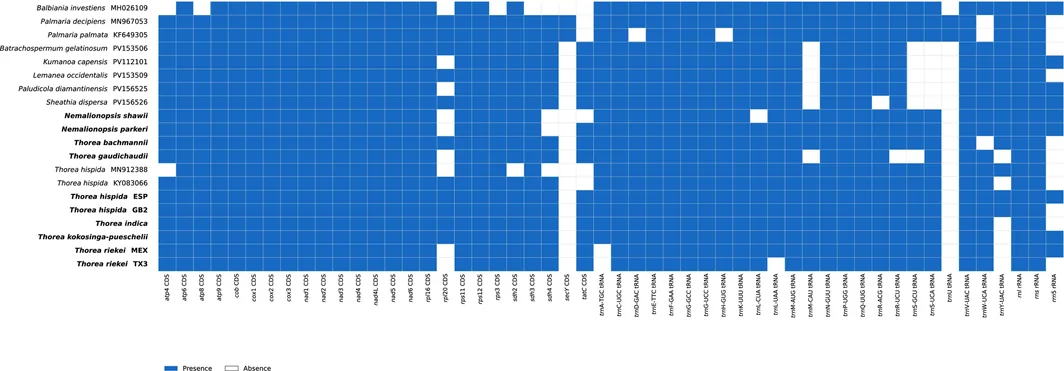
Binary heatmap of coding gene occurrence in the mitogenomes of taxa of Thoreales and related orders in Nemaliophycidae (Balbianiales, Batrachospermales, and Palmariales).

Intraspecific mitochondrial divergence was estimated as uncorrected pairwise *p*‐distances (calculated as 1 − sequence identity) from a whole‐mitogenome alignment using the MAFFT plug‐in (Geneious Prime® 2025.1.3) of four *Thorea hispida* individuals. Thoreales mitogenomes were aligned using the progressive MAUVE plug‐in (Darling et al., 2010) in Geneious Prime® 2025.1.3, applying the full alignment option, with automatically calculated seed weights and automated calculation of locally collinear blocks (LCBs) scored and compared to access genetic synteny.

## RESULTS

### Genome structure

Ten Thoreales mitogenomes were successfully de novo assembled and annotated and all were recovered in complete circular form (Figure S1). The mitochondrial architecture of all newly sequenced mitogenomes exhibited a high degree of conservation, displaying only minimal differences in size, gene content, gene number, and GC composition. Genome lengths ranged from 25,022 bp in *Thorea riekei* to 26,369 bp in *T. kokosinga‐pueschelii* (25,465 ± 387.3, overall mean ± standard deviation for all Thoreales taxa; Table 1). The total number of genes varied from 46 in *Nemalionopsis shawii* and *T. gaudichaudii* to 54 in *T. kokosinga‐pueschelii*. The set of protein‐coding genes (coding sequences, CDSs) ranged from 20 in *T. hispida* MN912388 to 25 in the three samples of *T. hispida*, *T. indica*, and *T. kokosinga‐pueschelii* (23.5 ± 1.73). The overall GC content varied between 25.6% in *T. kokosinga‐pueschelii* and 28.3% in *T. hispida* (all four specimens; 27.0 ± 1.0; Table 1). *Thorea gaudichaudii* had the lowest number of tRNA genes (21), and the highest (26) was observed for *T. kokosinga‐pueschelii* and *T. riekei* from Mexico (23.2 ± 1.47). The tRNA repertoire showed similar content among Thoreales species, except for the *trn*Y gene, which was present in only half of the mitogenomes. All species contained the rRNA genes *rrn*S (small subunit) and *rrn*L (large subunit), where the 5S rRNA gene was present in half of species: *N. parkeri*, *N. shawii*, *T. hispida* from Spain, *T. kokosinga‐pueschelii*, and *T. riekei* from Mexico (Figure 1).

**TABLE 1:** General features of the members of Thoreales mitogenomes generated in this study (bold) or previously published.

Taxa	Genome size (bp)	GC content (%)	Total gene number	Protein‐coding genes	tRNA; rRNA	Total gene length (bp)	% of intergenic region	Coverage (mean)
***Nemalionopsis parkeri***	25,222	27.5	48	22	23; 3	21,447	15.0	223
***Nemalionopsis shawii***	25,231	26.6	46	21	22; 3	21,997	12.8	1618
***Thorea bachmannii***	25,358	26.2	49	24	23; 2	23,751	6.3	202
***Thorea gaudichaudii***	25,868	26.8	46	23	21; 2	23,379	9.6	23.9
***Thorea hispida* ESP**	25,497	28.3	51	25	23; 3	24,301	4.7	1092
***Thorea hispida* GB2**	25,833	28.3	50	25	23; 2	24,188	6.4	1527
*Thorea hispida KY083066*	25,380	28.3	49	25	22; 2	24,079	5.1	‐
*Thorea hispida MN912388*	25,411	28.3	45	20	23; 2	22,189	12.7	‐
***Thorea indica***	25,354	28.2	50	25	23; 2	24,033	5.2	1302
***Thorea kokosinga‐pueschelii***	26,369	25.6	54	25	26; 3	24,494	7.1	198
***Thorea riekei* MEX**	25,029	26.3	47	23	21; 3	23,293	6.9	438
***Thorea riekei* TX3**	25,022	26.3	49	24	23; 2	23,331	6.8	973

The length of protein‐coding genes remained relatively conserved across the mitogenomes, with *atp*9 representing the shortest gene (~230 bp), and *nad*5 had the longest, reaching approximately 2000 bp. The proportion of intergenic regions ranged from 4.7% in *Thorea hispida* (ESP) to 15% in *Nemalionopsis parkeri* (8.2 ± 3.5). Intraspecific divergence among the four analyzed *T. hispida* mitochondrial genomes ranged from 0.6% to 2.1%, indicating moderate sequence variation among specimens from Asia and Europe. *Thorea riekei* specimens collected from Mexico and Texas exhibited divergence of 0.7%. Interspecific mitochondrial genome divergence within Thoreales varied substantially. Pairwise divergences among *Thorea* species ranged from 13.2%–32.0% across the genus. The two *Nemalionopsis* species exhibited a divergence of 16.2%, a value comparable to the lower limit observed for *Thorea*.

Among the Thoreales mitogenomes, *Thorea gaudichaudii* exhibited two premature stop codons: one in the *nad*2 gene and another in the *tat*C gene. *Thorea bachmannii* contained a premature stop codon in the *rps*11 gene, and *T. indica* harbored one in the *rpl*20 gene. Compared to the other Nemaliophycidae orders used as outgroups in this study, two gene losses were observed for all Thoreales mitogenomes: *sec*Y and *trn*U tRNA (Figure 1).

### Genome organization

ProgressiveMauve whole‐mitogenome alignment revealed a high level of structural conservation, showing only three LCBs shared among all Thoreales members. No rearrangements were detected, revealing a highly conserved syntenic organization in which both gene order and orientation were preserved (Figure 2).

**FIGURE 2:**
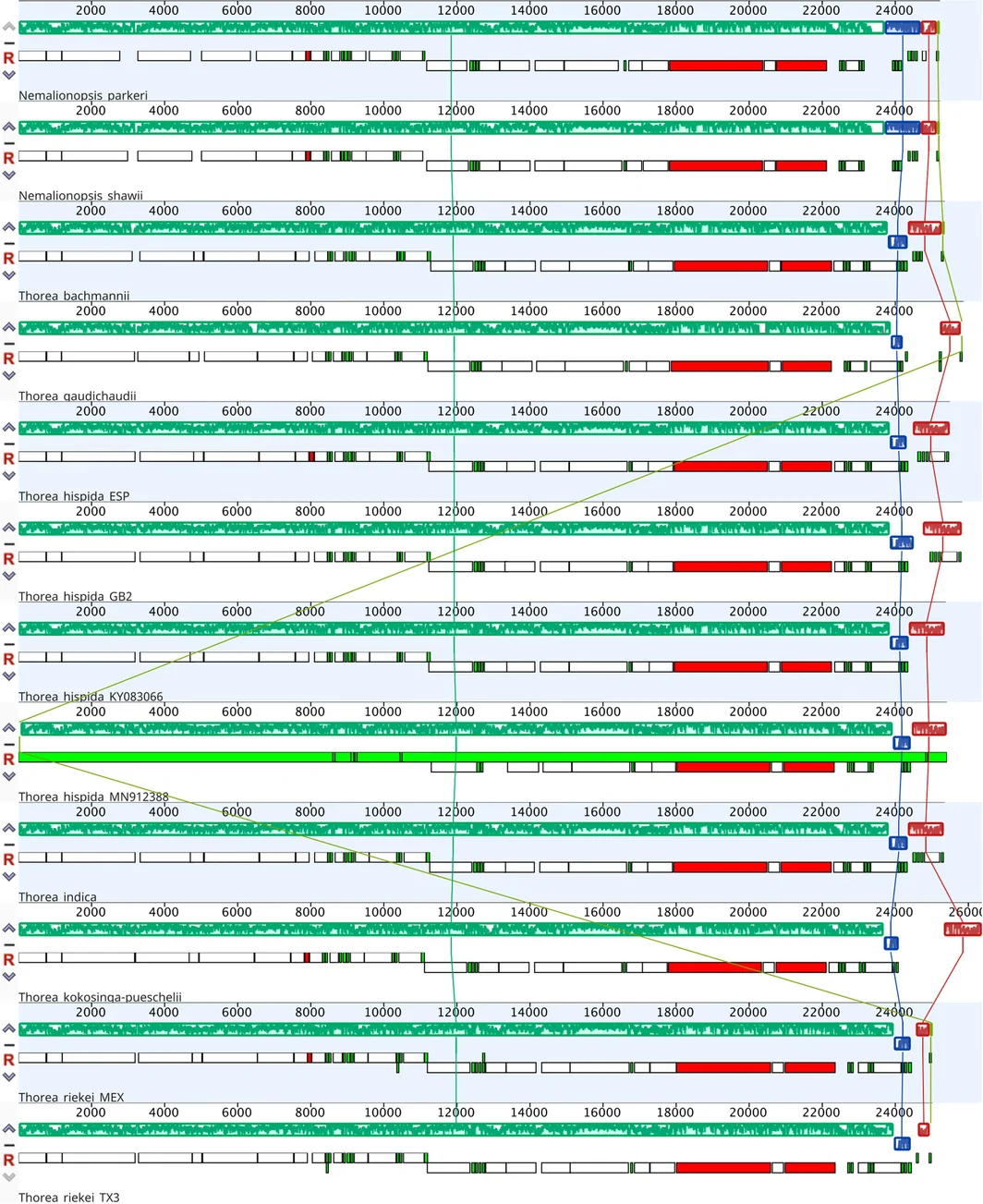
Synteny alignment of mitogenomes among taxa of the freshwater order Thoreales. Lines connecting each colored bar with a crossing “X” pattern represent genomic rearrangements or collinear structures annotated with different starting points. Two previously published mitogenomes of *Thorea hispida* (MN912388 and KY083066) were used in the alignment.

### Phylogeny

Phylogenomic analysis of concatenated mitochondrial dataset (16 CDSs) resulted in a maximum likelihood tree with 100% bootstrap support for the Thoreales clade, confirming it as a distinct lineage within the subclass Nemaliophycidae (Figure 3). Analysis also showed full support for all nodes within the order, except for two nodes within *Thorea hispida*. *Nemalionopsis* species were an early clade to *Thorea* species. Two clades were observed within *Thorea*: one with *T. gaudichaudii* from Oceania and *T. riekei* specimens from North America (the United States and Mexico) and another with *T. indica* and all four *T. hispida* sequences. Two species (*T. bachmannii* from South America and *T. kokosinga‐pueschelii* from North America) consisted of single lineages between these two clades.

**FIGURE 3:**
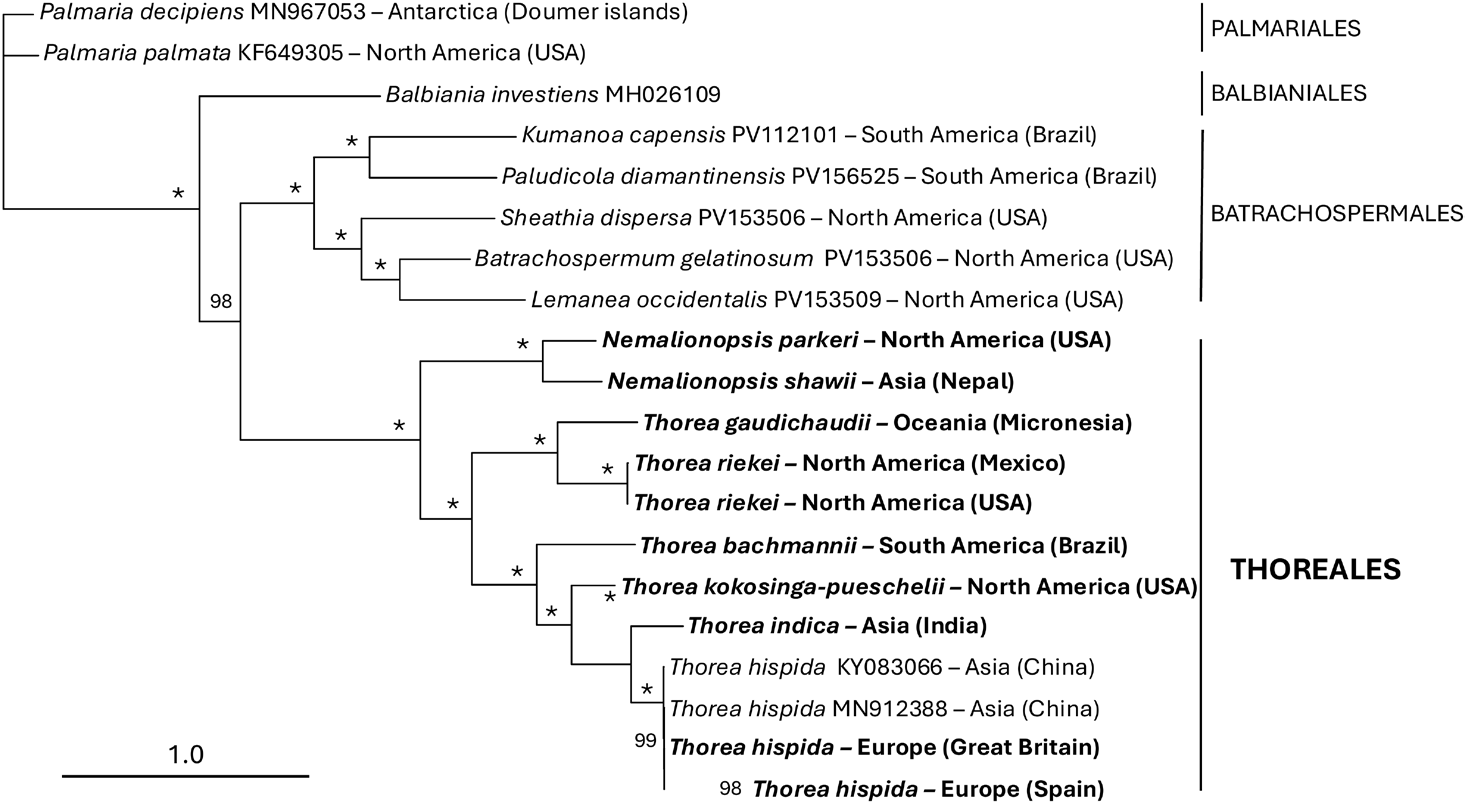
Maximum likelihood tree of the taxa of Thoreales with Balbianiales, Batrachospermales, and Palmariales as outgroups inferred from the concatenated alignment of 16 mitochondrial genes. Newly sequenced taxa are in boldface. Numbers along branches indicate bootstrap support, and nodes with full bootstrap support are indicated with an asterisk. The scale bar represents substitutions per site.

## DISCUSSION

Prior to this study, only two mitogenomes were available for Thoreales, both from *Thorea hispida* (Cho et al., 2018; Nan et al., 2017). With the present dataset, coverage increases to 10 mitogenomes representing both genera in the order and encompassing eight species. The features recovered for the newly sequenced species are consistent with previously published Thoreales mitogenomes and with patterns reported for other orders within Nemaliophycidae, particularly the exclusively freshwater order Batrachospermales (Lemes da Silva et al., 2025). Thoreales mitogenomes are gene dense and exhibit highly conserved gene orders, a condition also documented for other members of Florideophyceae (Kim et al., 2022; van Beveren et al., 2022; Yang et al., 2015). Nemaliophycidae includes exclusively freshwater lineages (Balbianiales, Batrachospermales and Thoreales) that typically exhibit compact, gene‐dense, and highly syntenic mitogenomes, a pattern clearly different from the freshwater *Riverina jigongshanensis* that stands out with the largest (42,459 bp) Florideophyceae mitogenome reported to date (Nan et al., 2023; Vieira et al., 2024). Although based on a limited number of freshwater lineages, this comparison suggests that freshwater colonization, per se, may not fully explain variation in mitochondrial genome size. Instead, differences in genome architecture may also reflect lineage‐specific evolutionary histories including patterns of intron gain, loss, and retention (van Beveren et al., 2022).

Comparative analyses of mitochondrial gene content showed that members of Thoreales share highly similar gene contents, underscoring the strong conservation of the mitochondrial gene repertoire within Nemaliophycidae (van Beveren et al., 2022; Yang et al., 2015). Notably, the *rpl*20 was absent from *Nemalionopsis parkeri*, *N. shawii*, *Thorea gaudichaudii*, *T. hispida* (MN912388), and both *T. riekei* specimens—a loss also observed in Balbianiales and some members of Batrachospermales (Lemes da Silva et al., 2025) and reported in other Florideophyceae mitogenomes (Kim et al., 2022; Salomaki & Lane, 2016; van Beveren et al., 2022).

The absence of several mitochondrial genes and the occurrence of premature stop codons in a subset of Thoreales taxa should be interpreted cautiously. Gene loss has been reported repeatedly across red algal organellar genomes and may result from multiple evolutionary processes, including functional transfer to the nucleus, pseudogenization, or lineage‐specific genome streamlining (Kim et al., 2022; Salomaki & Lane, 2016; van Beveren et al., 2022). However, distinguishing among these alternatives requires complementary genomic and transcriptomic evidence that is currently unavailable for most Thoreales species. Likewise, although the premature stop codons detected in the *nad*2, *tat*C, *rps*11, and *rpl*20 genes may indicate gene degeneration or ongoing evolutionary change, annotation uncertainty and assembly artifacts cannot be completely excluded. Given the overall conservation of mitochondrial genome architecture and gene content across Thoreales, these features are best regarded as candidate lineage‐specific modifications requiring further investigation rather than definitive evidence of functional gene loss.

Our analysis of the 12 Thoreales mitogenomes in relation to the three other orders of Nemaliophycidae revealed that three genome characteristics fell within the range reported for Batrachospermales but were closer to the lower values observed in Palmariales: genome size (29,735 bp vs. 25,022–26,369 bp), number of coding genes (26 vs. 20–25), and GC content (32.2% vs. 25.6–28.3%). In comparison, the mitochondrial genome of Balbianiales exhibits a larger size (26,389 bp) and higher GC content (31.6%) than all sequenced Thoreales species. However, both the total number of genes (44 vs. 45–54) and the number of protein‐coding genes (17 vs. 20–25) reported for Balbianiales fall below the lower limits observed in Thoreales.

Although these patterns could suggest differences in gene content or genome compaction among freshwater red algal lineages, such variation should be interpreted with caution, as discrepancies may also arise from annotation strategies, sequencing depth, or incomplete assemblies. Previous comparative studies of red algal mitochondrial genomes have shown that differences in gene counts frequently result from methodological inconsistencies rather than true gene loss or gain events (Muñoz‐Gómez et al., 2017; Salomaki & Lane, 2016; Yang et al., 2015).

In contrast to the variation observed in genome size and protein‐coding gene content, the differences detected in the tRNA repertoire suggest that these genomic regions are more prone to loss and rearrangement events. The absence of the *trn*A‐*TGC* tRNA gene in *Thorea riekei* specimens, together with variation in the *trn*S, *trn*Y, and *trn*W genes, may reflect functional replacement via cytosolic tRNA import, a mechanism documented in several red algal lineages (Alfonzo & Söll, 2009; Salinas et al., 2012). Greater divergence in the tRNA repertoires of the outgroup taxa (Balbianiales, Batrachospermales, and Palmariales) further supports the view that although the protein‐coding gene complement is largely conserved, the tRNA component displays considerable evolutionary plasticity. This plasticity may play an important role in ecological specialization and in maintaining populations under distinct environmental conditions (Cho et al., 2018).

Synteny among Thoreales mitogenomes was shown to be strongly conserved, with LCBs in identical order and orientation across all taxa and only minor intergenic/tRNA‐boundary length variation without large‐scale inversions.

Phylogenetic analyses of Thoreales, including the two currently recognized species of *Nemalionopsis* and six species of *Thorea*, showed high statistical support across all branches. At present, *Thorea* comprises 12 recognized species: nine supported by DNA sequence data and three described based on morphological characters or geographic distribution (Vis & Necchi, 2021). Although the present study included half of the currently recognized diversity within *Thorea*, the use of complete mitochondrial genome data provided a robust and well‐resolved phylogenetic framework. This work represents a mitogenome‐based phylogeny for Thoreales and establishes a solid basis for future studies incorporating broader taxonomic sampling. The resulting topology is consistent with earlier studies that relied on single‐ or few‐gene analyses (Johnston et al., 2018; Müller et al., 2002; Vis & Necchi, 2021). *Nemalionopsis* species were the earliest diverging lineage within the Thoreales, whereas two clades were recovered within the genus *Thorea* with no evident geographic or taxonomic association. Among the species of the genus *Thorea* sequenced here, *T. hispida* was the only one exhibiting a broad intercontinental range, with confirmed occurrences in Asia, Oceania, Europe, and North America (Johnston et al., 2018; Vis & Necchi, 2021). In this study, we included four *T. hispida* sequences: two previously published from Asia (both from China) and two new from Europe (Spain and Great Britain). Intraspecific divergence among these specimens ranged from 0.6% to 2.1%, indicating overall low genetic differentiation across continents. Divergence within the European samples was 1.3%, whereas Asian samples exhibited lower divergence (0.6%). Similarly, *T. riekei* specimens from two geographically distinct localities in North America (Mexico and Texas) showed low divergence (0.7%), close to the lower limit observed in *T. hispida*. Comparable values were also observed in the Batrachospermalean taxa from Asia, including *Kumanoa mahlacensis* with two specimens from China and Taiwan (0.3%) and *Sheathia dispersa* from Nepal and Taiwan (2.0%; Lemes da Silva et al., 2025). Taken together, these results indicate that mitochondrial genomes in both Thoreales and Batrachospermales exhibit relatively low intraspecific variability, even among geographically distant populations.

At the interspecific level, mitochondrial genome divergence within Thoreales was more pronounced. Among *Thorea* species, pairwise divergence (13.2% to 23.0%) exceeded those observed between the two *Nemalionopsis* species (16.2%), suggesting comparable levels of genomic divergence across both genera. The broader divergence range within *Thorea* likely reflects its higher species diversity as previously reported by Johnston et al. (2018), since the genus harbors more diversity than was previously recognized by morphological data alone. In addition, this divergence also indicates a deeper evolutionary diversification, while the overall similarity among Thoreales taxa supports a conserved mitochondrial architecture within the order. The level of intraspecific divergence is consistent with previous observations in freshwater red algae, wherein mitochondrial genomes tended to be conserved across wide geographic ranges (Lemes da Silva et al., 2025). Such patterns may reflect the slow evolutionary rate of red algal organellar genomes (Salomaki & Lane, 2016, 2019; Yang et al., 2015). Altogether, these factors may contribute to the maintenance of low mitochondrial variability within Thoreales species despite their broad geographic distribution.

This study provides the most extensive mitochondrial gene matrix for Thoreales to date, yielding a stable, well‐supported framework for relationships within the order and strengthening comparative context across Nemaliophycidae. Further progress on this group's evolutionary relationships will depend on broader sampling, particularly complete genomes from additional Nemaliophycidae orders, to allow for better‐supported hypotheses about interordinal affinities within Nemaliophycidae.

## AUTHOR CONTRIBUTIONS

**Monica O. Paiano:** Conceptualization (lead); data curation (lead); formal analysis (lead); investigation (lead); methodology (lead); writing – original draft (lead); writing – review and editing (lead). **Morgan L. Vis:** Resources (lead); writing – review and editing (equal). **Nadia M. Lemes da Silva:** Data curation (equal); formal analysis (equal); writing – review and editing (equal). **John A. West:** Funding acquisition (equal); resources (equal); writing – review and editing (equal). **Shiva K. Rai:** Resources (equal); writing – review and editing (equal). **Javier Carmona‐Jiménez:** Resources (equal); writing – review and editing (equal). **Orlando Necchi Jr:** Conceptualization (lead); funding acquisition (lead); methodology (equal); resources (lead); writing – original draft (equal); writing – review and editing (equal).

## FUNDING INFORMATION

This research was supported by FAPESP FJD Fellowship granted to MOP (Proc 2023/10342‐8) and by FAPESP grants to ON (Proc 2020/11064‐3, 2023/01404‐0).

## Supporting information


**Figure S1.** Gene map of the ten mitogenomes of taxa of Thoreales generated in this study. Genes shown on the outside of the circle are transcribed counterclockwise. Annotated genes are colored according to the functional categories shown in the legend at the bottom left. (A) *Nemalionopsis parkeri*. (B) *N. shawii*. (C) *Thorea bachmannii*. (D) *T. gaudichaudii*. (E) *T. hispida* GB2. (F) *T. hispida* ESP. (G) *T. indi*ca. (H) *T. kokosinga‐pueschelii*. (I) *T. riekei* MEX. (J) *T. riekei* TEX.

**Table S1.** Species, collection information, and GenBank accession numbers for mitochondrial genome sequences of Thoreales generated in this study.
