# Supplementary material for: Mitogenome structure and phylogenomics of the order Thoreales (Nemaliophycidae, Rhodophyta)
Source: J Phycol. 2026 Jul 23;62(5):1451–60. doi: 10.1111/jpy.70207 (PMC13659378; doi:10.1111/jpy.70207)
Supplement: Supplementary file 1 [file JPY-62-1451-s001.docx]

**Table S1.** Species, collection information, and GenBank accession numbers for mitochondrial genomes sequences of Thoreales generated in this study.

| **Species** | **Herbarium voucher** | **Collection information** | **GenBank Accession Number** |
| --- | --- | --- | --- |
| ***Nemalionopsis parkeri*** | CCAP 1376-1 | Small stream, in flowing water, immediately downstream from bridge Lower Barton Creek (type locality), Wake County, North Carolina, United States (35.971894, -78.634357). F.D. Ott. 12.XII.1973. | PZ303040 |
| ***Nemalionopsis shawii*** | SJRP 31583 | Khayer Khola, a small freshwater stream in Gurung Tol, Sundardulari-5, Morang District, Nepal (26.662376, 87.323709), O. Necchi Jr., ii.2018. | PZ303041 |
| ***Thorea bachmannii*** | SJRP 26892 | Campininha Stream, Bodoquena, Mato Grosso do Sul State, Brazil (-20.418333, -56.716944), O. Necchi Jr., 31.v.2002. | PZ303042 |
| ***T. gaudichaudii*** | BHO A-1236 | Wichen Falls, Weno, Chuuk, Federated States of Micronesia (7.463903, 151.854097), M.L. Vis, D.L. House, K. Kanto. 03.xii.2007. | PZ303043 |
| ***T. hispida*_GB2** | NA | Great Britain, Cambridgeshire, Houghton River, Hough Mill, 52.749762, -1.392934), J. Kwandrans, 21.ix.2010. | PZ303044 |
| ***T. hispida*_ESP** | BHO A-1342 | Rio Racons, Spain (38.882304, -0.037431). R. Andreu. | PZ303045 |
| ***T. indica*** | SJRP 31508 | Sai River, Uttar Pradesh, India (26.527252, 81.005724). K. Toppo.13.iv.2014. | PZ303046 |
| ***T. kokosinga-pueschelii*** | BHO A-1002 (isotype) | Kokosing River, Knox County, Ohio, United States (40.409900, -82.499272). J.K. Backus, et al. 12.ix.2011. | PZ303047 |
| ***T. riekei*_MEX** | NA | Santa Anita River, central Mexico (21°58´32´´ N, 99°11′ 28´´ W). J. Carmona, 29.iii.2025. | PZ303048 |
| ***T. riekei*_TX3** | BHO A-1339 | Comal Spring, Landa Park, Texas, United States. E.T. Johnston & M.L. Vis, 15.xii.2010. | PZ303049 |
